# Endoscopic replacement of a transgastric feeding tube in advanced oropharyngeal cancer with esophageal obstruction using transgastrostomy endoscopy

**DOI:** 10.1055/a-2764-4771

**Published:** 2026-01-13

**Authors:** Ahmed Alwali, Clemens Eissner, Imad Kamaleddine, Clemens Schafmayer

**Affiliations:** 1 39071 Department of General, Visceral, Thoracic, Vascular and Transplant Surgery, Rostock University Medical Center, Rostock, Germany

**Fig. 1:**
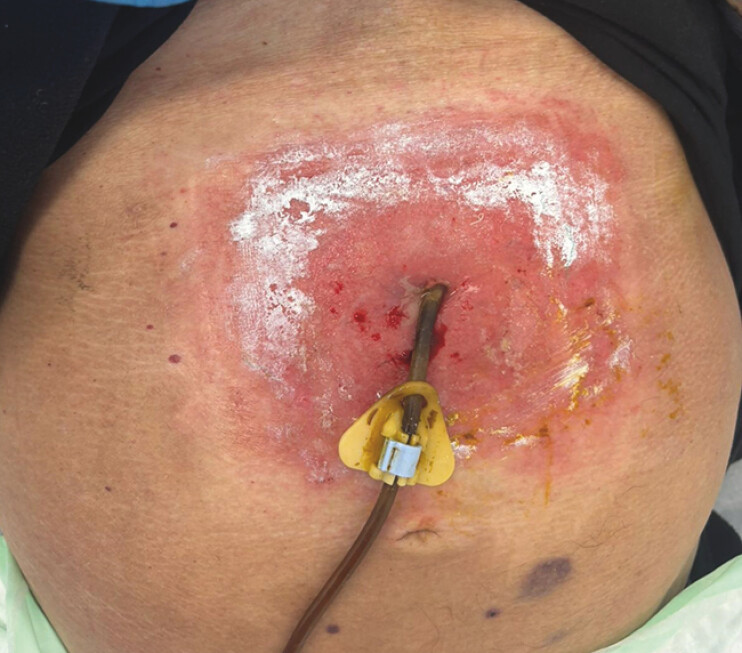
An infected gastrostomy site with purulent discharge and abdominal wall phlegmon.

A 78-year-old man with advanced oropharyngeal cancer presented with a therapy-resistant wound infection at the site of a previously placed percutaneous endoscopic gastrostomy (PEG). The infection required PEG-tube removal, performed using the cut-and-push technique. Owing to complete esophageal obstruction, conventional transoral endoscopic re-insertion of a new PEG was not feasible. ( Fig. 1 ).

**Fig. 2:**
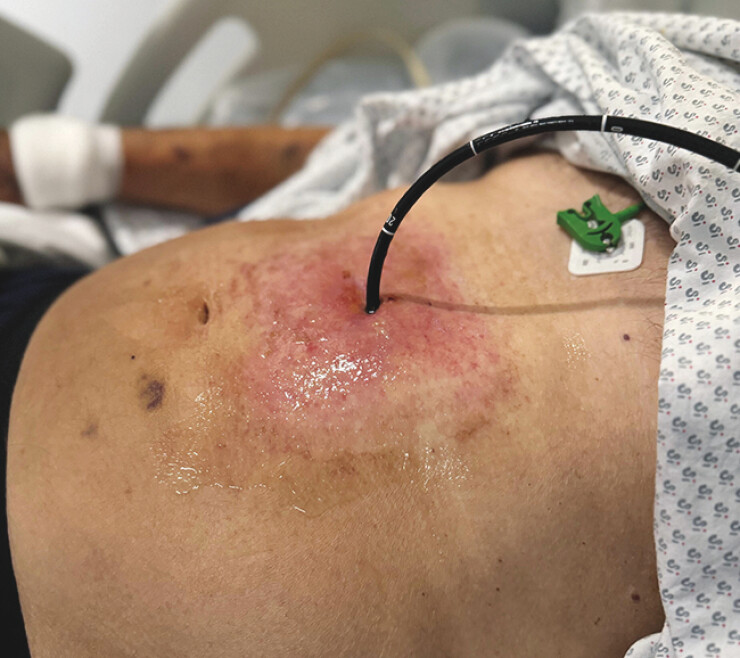
A trans-gastrostomy endoscopic approach using a nasal gastroscope through the gastrostomy tract to access the stomach.

A slim nasal gastroscope was advanced through the gastrostomy tract to access the stomach ( Fig. 2 ). Under direct endoscopic visualization, a new gastric puncture was performed in an unaffected region, followed by gastropexy and PEG reinsertion via the push technique. The procedure was carried out under sterile conditions without any complication ( Video 1 ).

A trans-gastrostomy endoscopic approach in a patient with complete esophageal obstruction. The nasal gastroscope was introduced via the gastrostomy tract, and a new PEG was safely placed under direct visualization.Video 1

Trans-gastrostomy PEG placement is a valuable alternative in patients with complete esophageal obstruction where conventional access is impossible. Careful patient selection and strict adherence to sterile technique enable the safe reinsertion of a PEG at a new gastric site, avoiding the need for surgical gastrostomy.

This case illustrates the feasibility and safety of trans-gastrostomy endoscopy for PEG replacement in complex anatomical situations and highlights its role as a minimally invasive salvage technique.

Endoscopy_UCTN_Code_TTT_1AO_2AK

